# Over-the-Counter Drug Misuse and Dependence: Public Health Ethics’ Foray into Fight against the Codeine Crisis

**DOI:** 10.3390/pharmacy10060155

**Published:** 2022-11-20

**Authors:** Hiroyasu Ino, Eisuke Nakazawa

**Affiliations:** Department of Biomedical Ethics, Faculty of Medicine, University of Tokyo, 7-3-1 Hongo, Bunkyo-ku, Tokyo 113-0033, Japan

**Keywords:** over-the-counter drugs, codeine, public health ethics, up-scheduling

## Abstract

Over-the-counter (OTC) drugs, such as codeine, are available as an OTC drug at common drugstores and major e-commerce platforms, despite their known propensity for causing psychological dependence and harm from overuse. Misuse of and dependence on over-the-counter drugs are serious public health issues. Possible measures include stricter regulation and control, as well as regulation of access to information and awareness activities, but their effectiveness is limited. Up-scheduling, in which OTC drugs are placed under prescription or full regulation, is expected to have a definite effect. However, up-scheduling poses public health ethical challenges. Up-scheduling restricts the freedom of consumers to purchase OTC drugs, and the economic right of manufacturers and sellers. Up-scheduling may also interfere with people’s right to seek self-care through the proper use of OTC drugs. Limited up-scheduling with respect to age may be an effective way to implement up-scheduling while avoiding ethical issues. However, from a public health perspective, it is necessary to improve the information infrastructure so that prescription drug information can be shared electronically, and to strengthen measures to prevent mental health problems among young people that lead to OTC drug misuse, even after up-scheduling.

## 1. Introduction

Over-the-counter (OTC) drugs can be purchased at pharmacies and drugstores without a doctor’s prescription; such a provision is expected to promote self-medication and reduce healthcare costs [1]. However, this amenity has also led to a serious public health crisis. While the active ingredients in OTC drugs may be effective in treating certain diseases, disorders, or symptoms, they may also have psychostimulant effects. The misuse of such drugs for intoxication has long been observed among users [2] and has thus been the subject of addiction therapies and policy worldwide [3,4]. These abused OTC drugs include codeine-based drugs, cough products such as dextromethorphan, sedative antihistamines such as diphenhydramine, decongestants and laxatives [5].

Among these kinds of OTC drugs, codeine, which is indicated for use as a cough suppressant and analgesic, is usually sold with laxity under the regulatory framework for addictive substances, in countries such as the United Kingdom, France, and Japan [5]. It is available as an OTC drug at common drugstores and major e-commerce platforms as well, despite its known propensity for causing psychological dependence and harm from overuse [6]. The wide availability of codeine has been especially detrimental in curtailing drug use among the youth. The average age of patients reported for acute poisoning or admission by OTC drugs, such as codeine, is about 20 years old, affecting the health of young people in particular. [7,8]. It thus occupies an important place in the discussion on OTC drug misuse and dependence.

In pharmacy practice, the ethical challenges of scheduling are complex [9] and seldom discussed comprehensively. Codeine is an opioid, so it has logically been discussed within the confines of “chronic pain” [10], although this view might be limiting [11]. Its non-medical use for its psychostimulant effect is also important, and this inspires us to conduct a public health ethics analysis of OTC misuse and dependence and public health interventions against it. To bolster our argument, we especially refer to cases in Japan and other countries.

## 2. Case Study Results

### 2.1. Case Study 1: Regulation of Codeine in Japan and Its Issues

The codeine OTC drug sales in 31 countries between 2013 and 2018 [12] show that what the Japanese spent per capita on codeine purchases ranked fourth, after Ireland, the United Kingdom, and Croatia. The increase in purchases per capita between 2013 and 2018 was the largest among the 31 countries, at over 1000%. Cases of poisoning among young people with antitussive expectorants, presumed to be cases of codeine poisoning, have increased since 2017 [7].

In Japan, the Ministry of Health, Labour and Welfare has issued a ministerial ordinance titled the *Regulation for Enforcement of the Act on Securing Quality, Efficacy, and Safety of Products Including Pharmaceuticals and Medical Devices* to control medical drugs. The section “Sales of Pharmaceuticals Suspected to Be Abused,” states that

When a pharmacy sells or provides pharmacy-made pharmaceuticals or OTC pharmaceuticals that are designated by the Minister of Health, Labor and Welfare as those suspected to be abused, etc. […], a pharmacy proprietor must conduct the same by any of the following methods.

This section orders pharmacists to verify the purchaser’s information and evaluate “the quantity recognized as necessary for ensuring the appropriate use of the pharmaceuticals before they sell” [1,13].

This ministerial ordinance is not without its flaws. First, it requires individual pharmacists to decide on the appropriateness of sales [14], which may be counter to regulatory effectiveness. While some pharmacists still firmly enforce the ministerial ordinance by enquiring about suspicious purchases and previous purchases at other pharmacies or drugstores, not all pharmacists adhere to this ordinance [14]. Therefore, patients seeking to misuse OTC drugs are likelier to use the services of pharmacies that do not take appropriate action. Although uniformity of regulations is important in public health interventions to prevent addiction, there are no penalties for violations of this section. It is unclear to what extent drugstores, which sell these OTC drugs for profit and generate revenue, will comply with the regulations. This scenario leaves issues of effectiveness unresolved. This is particularly problematic for online purchases, as it is difficult to take physical measures, such as putting OTC drugs behind the counter as in a brick-and-mortar pharmacy.

Second, if a purchaser strongly insists on the necessity of the drug on some grounds, it is expected to be difficult to refuse to sell the drug even if suspicion prevails. The psychological burden on pharmacists [15] associated with this is expected to be significant.

In the Japanese context, regulations on codeine are currently under consideration and are scheduled for public comment as of August 2022 [16]. However, this discussion is focused solely on lifting the restriction that had previously limited sales to liquid antitussives only. As long as the above ministerial ordinances remain in place, codeine misuse is expected to continue, even if the marketing restrictions are expanded to include common cold remedies.

### 2.2. Case Study 2: Up-Scheduling of Codeine in Various Countries

The legalization of a previously controlled substance (e.g., marijuana) or the conversion of a prescription drug to an OTC drug (e.g., steroids) is called down-scheduling, whereas the placement of an OTC drug under prescription or full regulation is called up-scheduling [17]. In some countries and regions, OTC drugs that have become a public health problem are up-scheduled into drugs that require a physician’s prescription. For example, codeine has been up-scheduled since 2010 in Canada, Australia, and parts of Europe. It was up-scheduled in Australia in 2018 and in some Canadian provinces in recent years. In Australia, after the 2018 up-schedule, there was a 50.8% decrease in poison center calls [18] and a 53% decrease in hospitalizations [19], while the use of other dependent drugs did not increase [20]. Similar trends have been observed in Canada and Ireland [21,22]. Such positive outcomes have made up-scheduling a popular public health intervention against OTC drug misuse, and federal up-regulation is proposed by Health Canada after these successful outcomes [23].

Up-scheduling includes various grades of regulation, such as bans on the use or requiring a prescription. In this study, we only discuss the requirement for prescriptions as the main subject of analysis.

## 3. Ethical Discussions of Up-Scheduling

While up-scheduling is effective, it has not been introduced globally because of issues in public health ethics.

### 3.1. Restrictions

#### 3.1.1. Freedom of Consumers to Purchase Commercially Available Products and Right to Do Wrong

Under economic freedom, consumers are free to purchase products that are commercially available within the scope of conformity with laws and regulations. As long as there is no harm to others, the right to do wrong even permits acts that are clearly harmful to one’s own health. However, John Stuart Mill states that a responsible agent who performs such acts must have an opportunity for responsible choice and self-determination as the principal ingredient of their own good [24,25]. Some argue [26] that warnings are sufficient for the principle of double effect (which will be discussed later). 

However, OTC drug misuse may not satisfy these premises for the following three reasons. First, OTC drug misuse, including codeine, is more common among young people [7], who tend to engage in risky behaviors attributed to high risk-seeking during adolescence [27]. Young people in this transitionary stage of development are less likely to make rational decisions. OTC drug misuse is also a coping mechanism to deal with psychological burdens and social difficulties when other therapeutic options and help are limited or the individual has a poor cognitive capacity to consider the risks of such behavior; that is, the authenticity of agency is problematic [28], which hinders rational, responsible decision-making.

Second, the dangers of OTC drug misuse may not be sufficiently known to consumers. Among misuse information spread through social-network services [12], there is a biased focus on the psychostimulant effects of OTC medications. The regulation of packaging of OTC drugs that may cause misuse has not been legislated, unlike tobacco for example. Most packaging only informs of misuse without nuance and detail. Consumers who expect a psychostimulant effect are likely not fully informed of the risk of addiction or acute poisoning [29], which may distort their choices. 

Third, once dependence is formed, the decision to use a substance is controlled by the consumer’s craving, which is the psychiatric characterstics of addiction. In this light, consumers who buy OTC drugs for non-medical use may not be granted the right to do wrong.

#### 3.1.2. Freedom of Manufacturer or Seller to Manufacture or Sell Unregulated Products

Manufacturers and sellers of OTC drugs positively contribute to patients’ access to healthcare resources and distribution of medication. Overall, they reduce the number of visits to healthcare facilities in accordance with the principle of self-medication.

However, the Japanese drug regulation law, the Act on Securing Quality, Efficacy, and Safety of Products Including Pharmaceuticals and Medical Devices in Japan, states that the purpose of the law is “to improve health and hygiene”, and as a means to achieve this goal, the law lists “preventing the occurrence or spread of health and hygiene-related hazards caused by the use of those pharmaceuticals” [30]. This means that it is necessary to address the unintended misuse of pharmaceuticals, an issue that manufacturers may not anticipate. Unlike luxury items such as tobacco or alcohol, pharmaceuticals are intended to improve health and hygiene, and their hazards are subject to regulation by law. The principle of double effect is used as an ethical justification when unintended adverse effects occur [31], and it has been used in the ethical analysis of general public policy [32], the adverse effect of drugs [33], and the down-scheduling of some regulated drugs [34]. The principle of double effect theory states that an act with an unintended effect is morally acceptable if the following criteria are met [31]:

1. The act itself must be morally good or at least indifferent.
2. The agent may not positively will the bad effect but may permit it. The bad effect is sometimes said to be indirectly voluntary.
3. The good effect must flow from the action at least as immediately (in the order of causality, though not necessarily in the order of time) as the bad effect. In other words, the good effect must be produced directly by the action, not by the bad effect. Otherwise, the agent would be using a bad means to a good end, which is never allowed.
4. The good effect must be sufficiently desirable to compensate for the allowing of the bad effect.

Self-medication with OTC drugs is itself a good practice that improves access to and distribution of medical resources, and thus satisfies criterion 1. As the good of self-medication is not seen through the effect of misuse, it also satisfies criterion 3. Although self-medication itself is not intended to promote misuse, it may not meet criterion 2 from the perspective of healthcare access and resource allocation if the treatment goal it aims to provide is achieved through other medications or treatments that are not addictive. For example, if OTC drugs are used for orthopedic and back pain, they can be alleviated through exercise or improved posture, and knee pain can be alleviated through weight loss. While OTC drugs can be used for acute cough, honey, a natural expectorant, is more effective among children, as a Cochrane systematic review has shown [35]. Honey is even more accessible and less economically burdensome than OTC medications. There are also more effective non-opioid combinations of simple analgesics [9] that are less addictive than codeine-based drugs. When considering criteria 4, the increase in dependence and the increase in acute poisoning owing to overdose are not enough to compensate for the reduction in the burden of hospital visits. Thus, criteria 4, the proportionality condition, is not met either. Therefore, the principle of double effect theory supports the up-scheduling of OTC drugs.

The argument here is based on the classical principle of double effect theory, which states that foreseeability is not intentional. If we take the position of Foot [36] and others that foreseeability and intention are indistinguishable, then allowing misuse to increase as a matter of public policy would be ethically much more unacceptable

### 3.2. Right to Health

OTC drugs have long been promoted as self-medication. Codeine may be arguably necessary for the right to health [37] when the quality of life is impaired by pain or cough. If codeine is up-scheduled, it will no longer be available at pharmacies without a medical doctor’s prescription.

However, non-codeine OTC antitussive medications do exist, as well as OTC medications with different medicinal ingredients that provide antitussive effects comparable to those of codeine and are not psychodependent [9]. Health promotion by individuals can be limited from a public health perspective and codeine does not need to be an OTC drug as long as it is available as a prescription drug.

### 3.3. Possible Measures and Ethical Issues

#### 3.3.1. Continued Marketing and Stricter Regulation and Control

In Japan, until 2022, only cough suppressants containing codeine were subject to regulation, while common cold remedies were not. In 2022, the subcommittee on drug safety of the Committee on Drug Safety in the Pharmaceutical Affairs and Food Sanitation Council will review the scope of the regulation; after public comments, all OTC drugs including codeine are expected to be placed under some marketing control measures [13]. However, although the number of OTC products covered has been expanded, there will be no change from the previous drug scheduling.

From the perspective of harm reduction, measures such as limiting the amount of drugs contained in one package, requiring an explanation at the point of sale, and placing the product behind the counter at the point of sale have been adopted [5], but their effectiveness is limited. It is also difficult for pharmacists to profile the risk of consumers [9,11]. The seller’s moral hazard is a further risk [38]. Given the psychopathological characteristics of dependence, such as cravings, the effectiveness of this measure is limited because it is highly likely that people will seek out and purchase from sellers who do not have adequate countermeasures in place.

#### 3.3.2. Regulation of Access to Information and Awareness Activities

As psychological distress and social difficulties [39] underlie addiction and misuse, public mental health interventions are important. However, it is difficult to completely eradicate addiction this way even if strong mental health solutions are available. For this reason, policy-based regulation of addictive substances must be enforced to effectively combat addiction. Some users start misusing OTC drugs because they are legally available and, unlike hard drugs, they have little psychological resistance. Therefore, it is important to raise awareness about OTC drug misuse. However, as seen in the case of alcohol dependence, there is a limit to the preventive effect of awareness-raising activities when the substance is widely available.

The information on the psychostimulant effects of OTC drugs is known to be disseminated through social networking sites [40]. In Japan, codeine is included in common cold remedies and is also subject to misuse, but the misuse of dextromethorphan has been less problematic by comparison. In the United States, however, dextromethorphan misuse became a problem in the early 2000s [13], and it has been a key abused OTC drug group whose trend is not observed in other countries [41]. Thus, there is a kind of epidemic in the distribution of information on OTC drug misuse among young people, and it is assumed that this is because of culturally dependent biases of information on social networking sites.

In some cases, codeine is processed by users to make its psychostimulant properties more potent [42,43]. This is similar to past cases of microwave processing [44] against misuse prevention, such as extended-release opioids. The dissemination of such information on websites and its wide accessibility through search engines is undesirable, as it increases the misuse of codeine and acute poisoning cases. One example to counter the wide availability of information on codeine preparation and use comes from mental health awareness strategies: individuals who seek information on self-harm are often met with suicide prevention hotlines on the first page of search engines. Although paternalistic, this could be a beneficial option that gives primacy to the correct information on OTC drug misuse. From the perspective of Mill’s principle of harm to others, the act of suggesting dangerous information can increase the risk of acute poisoning or dependence and can be considered harmful. According to Mill’s principle of utility, the rightness of an act or the validity of a rule should be guided by the criterion of utility. Therefore, the freedom of an organization or individuals to disseminate information to others that has certain negative effects is not consistent with the goal of promoting the overall social good, and it is considered to be limited by the harm principle [45,46]. Thus, there is a moral case for restricting access to harmful information.

However, some platforms such as Twitter only prohibit the selling and buying of and facilitating transactions in drugs and controlled substances [47]. As the purchase of OTC drugs is legal in itself, it is difficult to fully regulate the posting of information. Some social-network sites also offer advice from self-help parties seeking to recover from OTC drug misuse and dependence [48], and thus it is not desirable to uniformly regulate information on OTC misuse. It is necessary to discuss how and who should draw the line between harmful and useful information on various platforms.

#### 3.3.3. Up-Scheduling

Up-scheduling is an option when continuing sales, tightening regulations and controls, raising awareness, and regulating access to information fail to meaningfully curtail misuse of OTC drugs. Note that we only consider the up-scheduling OTC drugs to prescription drugs. In other words, the change is not a complete restriction of availability, but a requirement for a physician’s judgment. Thus, patients whose condition requires the drug can obtain it under their physician’s prescription; its availability will remain unchanged, while public health intervention will be minimal. Here, we further divide up-scheduling into two categories depending on the scale of the public health intervention.

**Limited up-scheduling**. In a cohort study of chronic cough among residents aged 20 years and older in Copenhagen [49], individuals with chronic cough were considerably older, with a peak in morbidity in their 60s. In a Danish cohort study [50] of individuals aged 45 years and older, the prevalence of chronic cough peaked in the 80s. Only a few cohort studies of cough in children have been conducted [51], and in a U.S. study of children younger than 18 years [52], 10.1% of all participants used cough and cold medications in the past week, but only 0.2% of all participants used codeine. Systematic reviews have concluded that OTC drugs for cough have little benefit in the symptomatic control of childhood cough [51].

Thus, it is likely that antitussive medications in minors and young adults are in lower demand than in older populations, and their effectiveness is questionable. When seen from the perspective of the codeine crisis among young people, the option of up-scheduling the drug for this demographic seems reasonable.

Regulating access to certain substances, especially in vulnerable populations, reflects the kind of public health intervention that is put into force for regulating alcohol and tobacco. This is a preferred intervention for codeine misuse because it minimizes the harm caused by codeine-based drugs while ensuring access and freedom for older populations, who are presumed to have the highest demand for self-medication. It is still unclear whether we can subdivide up-scheduling by age within the legal system of each country. Legal revisions may be necessary to adapt the law to country characteristics.

**Total up-scheduling**. One of the limitations of limited up-scheduling is that it cannot restrict the acquisition of codeine for misuse by persons outside the target demographic. It is not a thorough countermeasure against misuse and dependence among other age groups. Some misusers are codependent on others to obtain their addictive substances—a loophole in the policy of limited up-scheduling. Australia, Ireland, and some provinces of Canada as a result enforced complete up-scheduling without discrimination for age.

However, such a policy measure might present difficulties in access to the appropriate use of codeine [53]. Total up-scheduling should thus be complemented by providing information on appropriate access, alternative drugs with the same effect, and coping strategies. However, more regional studies are needed to examine the effect of up-scheduling on patients using codeine as indicated.

**Issues after up-scheduling**. As discussed above, the two types of up-scheduling are ethically acceptable and desirable. However, the following considerations must be considered when making these institutional changes:

First, a physician’s prescription does not entail the elimination of misuse. Codeine is an opioid that is subject to similar dependence issues as other prescription opioids and benzodiazepines. Physicians must rigorously evaluate the need for up-scheduled medications in each case and prescribe only the necessary doses. An information infrastructure must be established to enable the sharing of prescription drug information electronically to avoid doctor shopping and obtaining drugs from multiple clinics.

There is a possibility that codeine-dependent patients might suffer collateral harm from sudden withdrawal once the drug is up-scheduled [54], forcing pharmacists to violate the principle of nonmaleficence. Addiction treatment facilities must be prepared to handle new entrants into their detoxification programs before the up-schedule. The public should be educated about these detoxification programs, informed about the up-scheduling policy well beforehand, and prepare general drug stores to put this policy in effect.

Mental healthcare needs to be strengthened, especially for young people who are already engaged in or vulnerable to OTC drug misuse. If self-injury is a solitary coping behavior for suffering, OTC drug misuse is also a manifestation of young people’s attempts to survive difficult circumstances. If the coping skill needs addictive substances, and if OTC drug-related health problems are increasing year on year, we should continue to provide multifaceted support to young people, including support for limiting access to OTC drugs that carry a risk of death or disability.

## 4. Conclusions

While OTC drugs promote self-medication, release the burden on healthcare services, and improve access to medical resources, their increasing misuse has created a public health crisis that countries worldwide are responding to. Among OTC drugs prone to mass misuse, the right to do wrong—especially of young people and those who have become dependent—cannot be established under Mill’s principle. Further, sellers and regulators may not continue selling them over the counter because of the principle of double effect. From a viewpoint of public health ethics, these OTC drugs need to be up-scheduled as long as self-medication can be achieved through other less addictive alternatives. In doing so, less restrictive measures, such as limited up-scheduling for young people, may be considered.

## Data Availability

Not applicable.
